# Efficacy of *Lactiplantibacillus plantarum* PBS067, *Bifidobacterium animalis* subsp. *lactis* BL050, and *Lacticaseibacillus rhamnosus* LRH020 in the Amelioration of Vaginal Microbiota in Post-Menopausal Women: A Prospective Observational Clinical Trial

**DOI:** 10.3390/nu16030402

**Published:** 2024-01-30

**Authors:** Franco Vicariotto, Patrizia Malfa, Elisa Viciani, Federica Dell’Atti, Diletta F. Squarzanti, Andrea Marcante, Andrea Castagnetti, Rosetta Ponchia, Laura Governini, Vincenzo De Leo

**Affiliations:** 1 Humanitas-S. Pio X Hospital, 20159 Milan, MI, Italy; ginecologia@vicariotto.com; 2 R&D Department, SynBalance Srl, 21040 Origgio, VA, Italy; d.squarzanti@synbalance.care; 3 Wellmicro Srl, 40138 Bologna, BO, Italy; elisa.viciani@wellmicro.com (E.V.); andrea.marcante@wellmicro.com (A.M.); andrea.castagnetti@wellmicro.com (A.C.); 4 Immunomics Laboratory, Department of Health Sciences, Center for Translational Research on Autoimmune and Allergic Diseases (CAAD), University of Eastern Piedmont, 28100 Novara, NO, Italy; federica.dellatti@uniupo.it; 5 Department of Molecular and Developmental Medicine, University of Siena, 53100 Siena, SI, Italy; ponchia2@student.unisi.it (R.P.); laura.governini@unisi.it (L.G.); vincenzo.deleo@unisi.it (V.D.L.); 6 Medical Policlinic Colledoro, 53100 Siena, SI, Italy

**Keywords:** menopause, lactobacilli, vaginal microbiota, pro-inflammatory cytokines, probiotics, immunity, genitourinary syndrome of menopause, vaginal atrophy

## Abstract

The menopausal transition marks a significant physiological shift in women. Menopause-related symptoms can significantly affect a woman’s quality of life and probiotics have emerged as a promising avenue. This study aims to investigate the benefits of probiotics in improving vaginal well-being and microbiota composition in post-menopausal women. A prospective observational clinical trial was carried out enrolling 50 post-menopausal healthy women, aged between 45 and 65 years old, taking a supplement containing *Lactiplantibacillus plantarum* PBS067, *Bifidobacterium animalis* subsp. *lactis* BL050, and *Lacticaseibacillus rhamnosus* LRH020 (3B CFU/day) for 28 days. Vaginal swabs were collected to evaluate microbiota fluctuation and the inflammatory pattern was recorded. A Vaginal Health Index was provided to evaluate vaginal well-being throughout the trial. Clinical outcomes revealed a decrease in menopausal symptoms. Significant improvements were observed across various parameters: a 50% enhancement in the VHI score (*p* < 0.0001), alongside substantial reductions in inflammatory cytokine levels. An 87.8% decrease in IL-6, 57.6% in IL-1β, and 40.8% in TNF-α was observed (*p* < 0.05). Moreover, the probiotic intervention facilitated the restoration of vaginal microbiota, evidenced by an increase in lactobacilli abundance. In conclusion, the combination of these specific probiotic strains, previously clinically tested in childbearing-age women, showed to be effective also for post-menopausal women.

## 1. Introduction

Menopause is a physiological phase in a woman’s life corresponding to the end of menstrual cycles and the reduction of ovarian function, which leads to a drastic decrease in oestrogen and progesterone levels as well as bio-psychosocial changes [1,2]. The onset of menopause is typically observed in women between 45 and 55 years, with an average age of 51 [3,4]. In some cases, this condition may either be caused or enhanced by surgical removal or permanent damage of the ovaries due to treatment with anti-cancer drugs [5]. According to the current life expectancy, it can be assumed that most women will spend almost 40% of their lives in menopause.

The decrease In oestrogen can cause different disorders and symptoms, both neuro-vegetative (hot flushes, profuse sweating, palpitations, tachycardia, blood pressure fluctuations, sleep disturbances, vaginal dryness, and genital itching) and psycho-affective (irritability, unstable mood, fatigue, anxiety, demotivation, concentration and memory disturbances, and decreased sexual desire) [1]. Another consequence is the increase in the frequency of urinary tract disorders, as often happens in the transition to menopause [6]. Different studies on menopausal women have also shown that low oestrogen levels can lead to significant anatomical alterations, such as changes affecting the genital area and vagina, resulting in an increased incidence of irritating genital symptoms [7]. Furthermore, oestrogen deficiency results in the thinning of the vaginal mucosa and the reduction of vascularisation, with a consequent loss of elasticity and hydration. The combination of these factors leads to a shift in the vaginal microbiota with a reduction in the lactobacilli genera, an increase in the pathogenic species, and a consequent rise in the vaginal pH [8]. In addition, it has been widely demonstrated that oestrogen plays a crucial role in the production of glycogen by stimulating the proliferation of squamous epithelial cells. The glycogen metabolism leads to the bioavailability of glucose, maltose, and other carbon sources, which are essential for the growth of lactobacilli [9].

Lactic acid bacteria (LAB), especially lactobacilli, can act by rebalancing vaginal homeostasis through different mechanisms: enhancing the epithelial barrier function, colonising the vaginal epithelium, inhibiting the adhesion capacity of pathogens, reducing the vaginal pH, producing antimicrobial substances, and, most importantly, increasing mucosal immunity and vaginal well-being [10]. In vitro tests demonstrated that *L. plantarum* PBS067, *L. rhamnosus* LRH020, and *B. animalis* subsp. *lactis* BL050 were able to exert anti-aggregation activity against the most common urogenital pathogens [11]. In addition, childbearing-aged women taking a food supplement containing the same strains reported a beneficial effect in the restoration of the vaginal microbiota as well as in the low recurrence rate of bacterial vaginosis, thanks to vaginal colonisation by oral intake [12]. Currently, there is strong evidence that several diseases pertaining to the urogenital area, including recurrent urinary tract infections (UTIs), bacterial vaginosis (BV), *Candida* vulvovaginitis (VVC), and aerobic vaginitis (AV), are related to an imbalance of the vaginal microbiota, called vaginal dysbiosis [13]. By studying the vaginal composition in different populations, it was possible to group women of childbearing age into five different categories, identified as Community State Types (CSTs). The group of women with a lower incidence of infectious diseases, and therefore considered healthy, is identified as CST I. In this group, lactobacilli are the predominant microorganisms, among which the most prevalent is *Lactobacillus crispatus*. In the case of BV, the associated group is CST IV, characterised by the depletion of lactobacilli and the presence of several species of anaerobic pathogens (CST IV A) or *Atopobium* and *Megasphaera* (CST IV B) [14]. Moreover, lactobacilli, and in particular *L. crispatus*, are referred to as protective agents of atrophic vaginitis, as their absence predisposes to the condition corresponding mainly to the CST-IV group [15].

In summary, all the physiological changes induced by the reduction of oestrogen that occurs during menopause lead to vaginal dysbiosis, mainly characterised by the significant and progressive reduction of lactobacilli, which often causes vaginal infections. All these events might trigger an inflammatory response with the consequent increase of pro-inflammatory cytokines, such as interleukin (IL)-1β, tumour necrosis factor (TNF)-α, IL-6, IL-8, and leukocytes [16].

Different studies clarify how the use of specific probiotic products can restore the physiological vaginal microbiota, but they are mainly carried out on childbearing-age women, affected by BV or VVC [17]. In fact, oral and topical probiotics have been extensively studied in women of reproductive age, whereas the menopausal phase has not been deeply investigated yet [18]. Hence, studying the role of the microbiota in healthy menopausal women could help improve strategies for the modulation of the microbiota and prevent possible dysfunctions [19,20].

Nowadays, one of the current treatment options is hormonal replacement therapy (HRT) to restore the production of sexual hormones, including oestrogen. This is not suggested for all menopausal women since it has some side effects (headache, vaginal bleeding, and leg cramps) and can increase the risk of breast cancer and heart disease [21]. Considering that probiotics can influence not only the onset of troublesome infections but also the dysfunction of bone, adipose, or other tissues, such probiotic-based treatments might be a solid alternative to HRT and might be implied as a preventive strategy [19,22].

This prospective observational clinical trial aims to evaluate the efficacy of a food supplement, containing a specific probiotic formulation of *Lactiplantibacillus plantarum* PBS067, *Bifidobacterium animalis* subsp. *lactis* BL050, and *Lacticaseibacillus rhamnosus* LRH020, against the onset of vaginal dysbiosis, typical during menopause, and the establishment of inflammation due to vaginal infections.

## 2. Materials and Methods

### 2.1. Study Design

This prospective observational clinical study was carried out at the Medical Colledoro Center (Siena, Italy) in collaboration with the Department of Molecular and Developmental Medicine of the University of Siena (Siena, Italy), and the Department of Health Science of the University of Eastern Piedmont (Novara, Italy). This study was conducted in accordance with the principles of the Declaration of Helsinki and its later amendments or comparable ethical standards. Informed consent was obtained from all the participants enrolled before the beginning of the clinical study. All procedures involving human subjects were approved by the Independent Ethics Committee of Derming (Milan, Italy). The study was recorded at the ISRCTN registry with the following number: ISRCTN15737648.

Fifty healthy women who were aged between 45 and 65 years old and in menopause for at least 18 months were enrolled. The enrollment lasted from September 2022 to February 2023.

The use of oral antibiotics, the consumption of probiotics in food supplements or functional foods within 30 days before the screening visit, and HRT were considered exclusion criteria. The complete list of the inclusion and exclusion criteria is reported in Table 1.

### 2.2. Intervention

**Figure 1:**
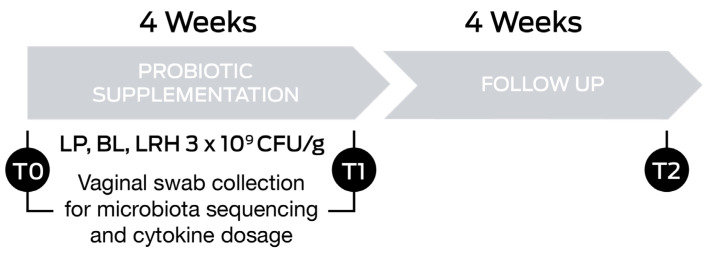
Timeline of the study.

Subjects were supplemented with a probiotic product containing *Lactiplantibacillus plantarum* PBS067 (DSM 24937), *Bifidobacterium animalis* subsp. *lactis* BL050 (DSM 25566), and *Lacticaseibacillus rhamnosus* LRH020 (DSM 25568; all from SynBalance Srl, Origgio, Italy) and manufactured by ROELMI HPC Srl (Origgio, Italy). Each capsule contained 3 × 10^9^ CFU/g (1 × 10^9^ CFU/g of each probiotic strain), vitamin B3, and maltodextrin. Women were asked to consume 1 capsule/day, away from meals, for 4 consecutive weeks. Assessments of health status were carried out at the starting point of the study (T0), after 4 weeks of oral probiotic supplementation (T1), and after a 4-week follow-up (T2; Figure 1).

Treatment efficacy was evaluated at T1 and T2. Compliance was evaluated at the follow-up visit when the subjects reported their experience. Vaginal swab samples were collected from the posterior fornix by a gynaecologist at the enrollment (T0) and the end of the probiotic treatment (T1) by using eNAT^®^ and eSwab^®^ collection systems (both from Copan Italia SPA, Brescia, Italy) for vaginal microbiota and cytokines analysis, respectively. The former was used to extract genomic bacterial DNA to be sequenced, while the latter was used to determine the concentrations of cytokines (Figure 1). After collection, samples were immediately transferred to the laboratory, processed, and stored at −80 °C.

### 2.3. Endpoints and Outcomes

The primary endpoint was the evaluation of the efficacy of a probiotic food supplement in improving vaginal dysbiosis by increasing lactobacilli and inhibiting the growth of pathogenic microorganisms in the vaginal microbiota.

The secondary efficacy endpoints were the assessment of functional well-being by the Vaginal Health Index (VHI), a reduction of the vaginal pH, and a decrease in pro-inflammatory cytokine levels (IL-6, IL-1β, TNF-α, and IL-8) in the vaginal samples.

### 2.4. Next Generation Sequencing

#### 2.4.1. DNA Extraction and Purification

Total microbial DNA was extracted from vaginal samples using the DNeasy 96 PowerSoil Pro QIAcube HT Kit on the QIAcube HT instrument (QIAGEN, Hilden, Germany) following the manufacturer’s instructions. A bead-beating step with Lysing Matrix E (MP Biomedicals) was performed on a FastPrep24 bead-beater (MP Biomedicals, Irvine, CA, USA) at 6.0 movements per second for 40 s, before total DNA extraction. Negative controls were PCR-grade water which underwent library preparation steps and Next Generation Sequencing (NGS) along with all the other samples. DNA was quantified using the Qubit™ 4 Fluorometer (Fisher Scientific Italia, Segrate, Italy).

#### 2.4.2. Determination of Bacterial Profiles by Amplicon Sequencing

The V3 to V4 region of the 16S rRNA gene was amplified using the primer set S-D-Bact-0341-b-S-17/S-D-Bact-0785-a-A-21 [23]. Indexed libraries were prepared by limited-cycle PCR using Nextera technology (Illumina, San Diego, CA, USA) and further cleaned up with VAHTS DNA Clean Beads (Vazyme, Red Maple Hi-tech Industry Park, Nanjing, China). The libraries were pooled at equimolar concentrations (4 nM), denatured, and diluted to 5 pM before loading onto the MiSeq Reagent kit V3 (Illumina). Sequencing on the MiSeq platform was performed by using a 2 × 300 bp paired-end protocol, according to the manufacturer’s instructions (Illumina). The raw sequence files were deposited in the Sequence Read Archive (SRA) under the project number PRJNA1054360.

#### 2.4.3. Data Processing and Analysis

Patients who either did not collect samples at the starting point of the study or at the end were dropped from the analysis. Therefore, the sequenced reads of 43 patients’ gut microbiota were analysed using QIIME2 (version 2020.6) [24]. The DADA2 (Divisive Amplicon Denoising Algorithm 2) plugin was used to remove noise and chimeras and to generate ASVs (Amplicon Sequence Variants) [25]. Quality filtering and clustering were performed using VSEARCH 2020.6.0 [26]. High-quality reads were classified taxonomically using the SILVA reference database version 132 [27]. The taxonomic classification of each Lactobacillus ASV was confirmed by aligning ASV’s sequence to NCBI Nucleotide Collection (nr/nt) against the Lactobacillus database (tax id: 1578) using the online Nucleotide BLAST program (https://blast.ncbi.nlm.nih.gov/Blast.cgi?PROGRAM=blastn&BLAST_SPEC=GeoBlast&PAGE_TYPE=BlastSearch, accessed on 4 April 2023). The data were imported into R (version 4.2.2) (R Core Team, 2022) on Rstudio (RStudio Team, 2020, version 2022.07.2 Build 576), where downstream analysis was performed using the R packages phyloseq, rbiom, ggplot2, tidyverse, tidyr, vegan, ape, ggpubr, and dplyr [28,29] (https://cran.r-project.org/web/packages/vegan/vegan.pdf; https://link.springer.com/book/10.1007/978-3-319-24277-4, accessed on 22 December 2023; https://dplyr.tidyverse.org/, accessed on 22 December 2023). Environmental microbial contaminants were excluded from the present analysis by filtering out ASVs that were specifically present in the negative controls using the decontam R package at 5% stringency [30]. Normalisation by rarefaction without replacement was performed to correct for different sequencing depths of each sample at 6643 reads, where the samples reached the maximum total ASV number asymptote. The differences in alpha diversity were evaluated, based on the data distribution of metrics, using ANOVA and Tukey’s HSD (honestly significant difference) tests for normally distributed data or the Wilcoxon–Mann–Whitney with the Holm–Bonferroni correction method (WMW with HB) for non-normally distributed data. To compare the microbial composition between samples, beta diversity was measured by calculating the weighted or unweighted UniFrac distance matrix on bacterial data [31]. Principal coordinates analysis (PCoA) was applied on the distance matrices to generate bi-dimensional plots in R. Dispersion of the PCoA clusters was compared using the betadisper function in the R vegan package [32]. The permutational analysis of variance (PERMANOVA) test, calculated using the function adonis2 in the vegan package, was performed to determine whether there was a significant separation between different sample groups (https://cran.r-project.org/web/packages/vegan/vegan.pdf, accessed on 22 December 2023). The linear discriminant analysis (LDA) effect size (LEfSe) algorithm, a tool which is hosted on the Galaxy web application at https://huttenhower.sph.harvard.edu/galaxy/ (accessed on 22 December 2023), was used to discover bacterial taxa associated with the treatment [33]. The differences in abundance were regarded as significant when the logarithmic LDA score was higher than 2. The alluvial plot was produced using the ggalluvial R package, while the heat map of the hierarchical clustering was obtained using the pheatmap R package (https://corybrunson.github.io/ggalluvial/articles/ggalluvial.html, accessed on 22 December 2023; https://cran.r-hub.io/web/packages/pheatmap/index.html, accessed on 22 December 2023).

### 2.5. Vaginal Health Index

The VHI is a clinical tool which evaluates five parameters (vaginal hydration, elasticity, secretions, pH, and epithelial mucous membrane) to assess the condition of vulvovaginal atrophy (VVA) and the well-being of the vagina [34]. The vaginal health is measured by the grading of each parameter (from 1 to 5), with a final score ranging between 5 and 25, with a cut-off <15 as an index of a VVA environment [35]. The parameters considered and the related scores are reported in Table 2.

### 2.6. Cytokine Concentrations

The concentrations of IL-6, IL-1β/IL-1F2, IL-8/CXCL8, and TNF-α in the vaginal swab samples were determined by using a Duo Set^®^ Kit (R&D Systems, Minneapolis, MN, USA distributed by Bio-Techne, Milan, Italy) according to the manufacturer’s instructions. Briefly, 100 μL of standards and diluted samples were added in duplicate wells in a 96 well-plate (VWR International Srl, Milan, Italy) coated with the primary antibody (Thermo Fisher Scientific, Waltham, MA, USA) and incubated for 2 h (h) at room temperature (RT). After the washing steps, the secondary antibody was added, and the plates were incubated for 2 h at RT. Streptavidin-HRP was added after washing and incubated for 20 min (min) at RT, avoiding direct contact with light. Then, the substrate solution (TMB ready-to-use solution, Sigma-Aldrich, St. Louis, MO, USA distributed by Merck Life Science Srl, Milan, Italy) was added to each well, and the stop solution was added after an incubation of 20 min at RT (Stop Solution, BioLegend, Houston, Texas, USA distributed by Campoverde Srl, Milan, Italy).

The absorbance was measured at 450 nm using a Spark microplate reader (Tecan Group Ltd., Männedorf, Switzerland) and the concentration of inflammatory markers was calculated using a standard curve (pg/mL).

### 2.7. Statistical Analysis

The results reported in this paper are for the per-protocol (PP) population and include all the subjects with complete data for all the endpoints.

The Student *t*-test for paired data was used to determine whether there was a statistically significant variation over time in the analysed endpoints. All the statistical analysis was one-sided at a 5% significance level (*p* < 0.05) and conducted by NCSS 10 (version 10.0.7 for Windows; NCSS, Kaysville, UT, USA) running on Windows Server 2008 R2 Standard SP1 64-bit edition (Microsoft, WA, USA). The level of significance was reported as follows: * *p* < 0.05, ** *p* < 0.01, *** *p* < 0.001, and **** *p* < 0.0001.

#### NGS Statistical Analysis

A permutational multivariate analysis of variance (PERMANOVA, 999 permutations) was used to test the difference among groups of microbial beta diversity. Differentially abundant taxa were identified by linear discriminant analysis (LDA) effect size (LEfSe). Categorical variables are presented as counts and percentages, and continuous variables as median, minimum, and maximum values. For group comparisons, Shapiro–Wilk’s test or Kolmogorov–Smirnov Test of Normality was used to test data for normality assumptions. Fisher’s exact test was used to analyse categorical variables, the Mann–Whitney U test was used on non-normally distributed continuous data, and the *t*-test was performed on normally distributed continuous data.

## 3. Results

All women involved in the study well tolerated the food supplement treatment; indeed, no adverse events and only one dropout, independent of the treatment, were reported throughout the study. The clinical study included a total of 49 subjects, all residing in Tuscany, Italy. The mean age of the women enrolled was 59.80 ± 6.41 years with a mean body mass index (BMI) of 23.79 ± 2.70 and a mean number of deliveries of 1.45 ± 1.02. Eight subjects were smokers, none received HRT or had comorbid conditions due to type 1 or 2 diabetes. Only one woman consumed yoghurt during the study.

### 3.1. Vaginal Microbiota by NGS

Samples were collected at the beginning of the study (T0) and the end of the treatment (T1). Forty-three out of forty-nine vaginal samples were analysed since some of them failed to produce a valid sample (missing sample).

**Figure 2:**
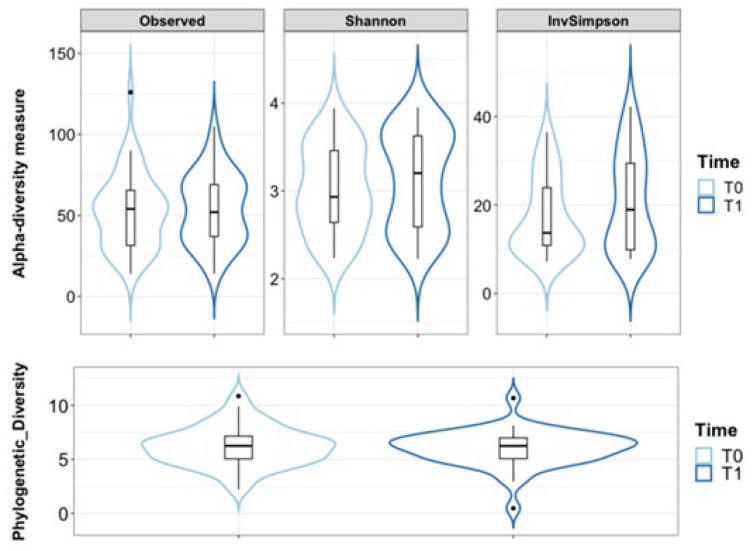
Violin plots with box-and-whisker plots showing the comparison of alpha diversity measures between menopausal women at the beginning (T0, *n* = 43, in light blue) and at the end of the probiotic treatment (T1, *n* = 43, in blue). Observed = Observed Species; Phylogenetic_Diversity = Phylogenetic Diversity Whole Tree; Shannon = Shannon–Wiener index; InvSimpson = Inverse Simpson’s index. Median, first, third quartile and outliers are shown.

Alpha diversity measures were used to summarise the distribution of species richness and evenness in each sample. The Observed Species diversity index, which counts the unique ASVs (Amplicon Sequence Variants) that are present in each sample, expressing the richness of the ecosystems, showed that in our data the vaginal ecosystems remained similar at the two experimental time points (Figure 2).

The Shannon–Wiener index and the Inverse Simpson’s index, which evaluates both richness and evenness, were assessed (Figure 2). The Shannon–Wiener index places more emphasis on rare species, while the Inverse Simpson’s index assigns more weight to dominant species [36]; the values of both indexes demonstrated similar results at the beginning and the end of the study. Moreover, the analysis of the phylogenetic relationships among taxa in each subject showed that post-menopausal women had a similar phylogenetic diversity before and after the treatment (FDR-corrected *p* = n. s.; Figure 2). These results indicate that the vaginal ecosystem was not perturbed by the probiotic treatment and kept its original richness and evenness of distribution of bacterial taxa.

**Figure 3:**
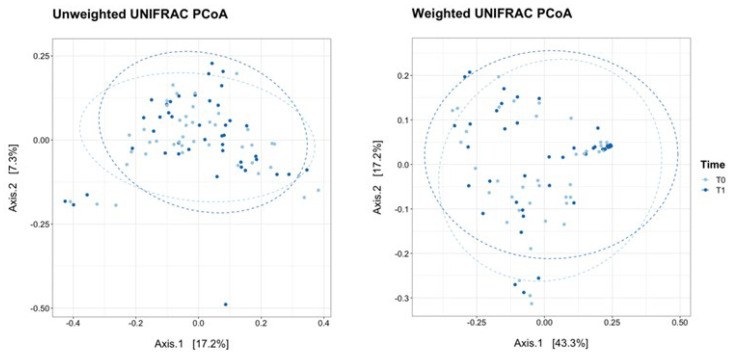
Principal Coordinate Analysis (PCoA) on unweighted and weighted UniFrac distance metric calculated on menopausal women at T0 (*n* = 43, light blue dots) and T1 (*n* = 43, blue dots). Each sample is represented by a dot. Axis 1 explained 17.2% and 43.3% of the variation observed, while Axis 2 explained 7.3% and 17.2% of the variation, in the left and right graph, respectively; dashed light blue ellipses, for T0 data, or blue ellipses, for T1 data, were calculated on the cluster of the sample data using the function ‘stat_ellipse’ and assuming a multivariate t–distribution; PERMANOVA on weighted and unweighted UniFrac Pr(>F) = n. s. (not significant).

The principal coordinate analysis (PCoA) revealed that the vaginal microbiota of post-menopausal women was similar throughout the trial (Figure 3).

Indeed, the weighted and unweighted UniFrac measures displayed no significant clusterisation between T0 and T1 (PERMANOVA Pr(>F) = n. s.).

**Figure 4:**
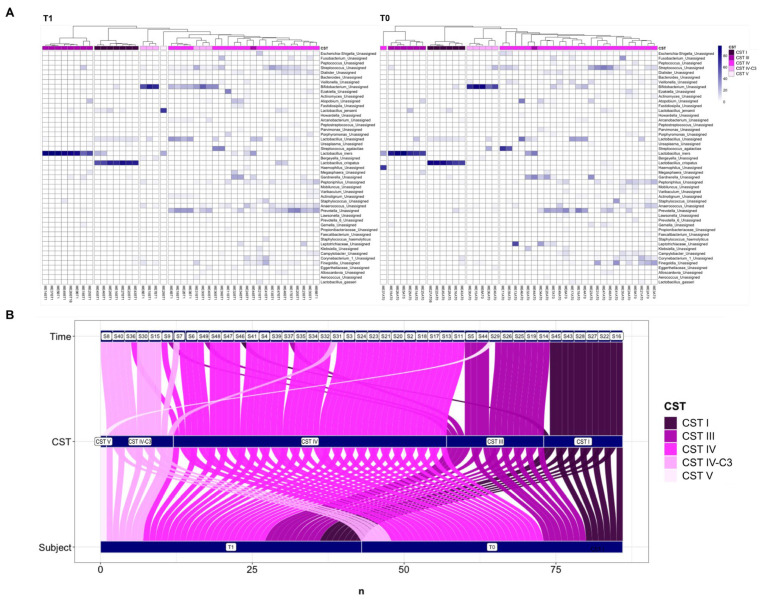
Heat map of the core microbiome in the vaginal samples based on hierarchical clustering of bacterial species relative abundance (RA%) with an alluvial plot of the Community State Type changes in time. RA% color scale is indicated on the right with white corresponding to 0% and dark blue corresponding to 100%. (**A**) The vaginal microbiota at T0, on the right, can be divided into 4 clusters, corresponding to 4 different Community State Types (CSTs): CST I (*n* = 6), CST III (*n* = 7), CST IV (*n* = 25), and CST IV-C3 (*n* = 5). The vaginal microbiota can be divided into 5 clusters at T1, on the left: CST I (*n* = 7), CST III (*n* = 9), CST IV (*n* = 20), CST IV-C3 (*n* = 6), and CST V (*n* = 1). (**B**) Alluvial plot of the CST changes per subject of the study before (T0) and after treatment (T1).

At the beginning of the experiment, our dataset of the vaginal ecosystems was divided into four Community State Types (CSTs): six patients were dominated by *Lactobacillus crispatus* (CST I), seven were dominated by *L. iners* (CST III), 25 contained a low proportion of lactobacilli and were composed of a polymicrobial mixture of bacteria (CST IV), while five belonged to the recently characterised CST IV-C3, where Bifidobacterium was the dominant taxon [37]. At the end of the experiment, the vaginal ecosystems were divided into five clusters: seven patients belonged to CST I, nine to CST III, 20 to CST IV, six to CST IV-C3, and one to CST5, the CST dominated by *L. jensenii* (Figure 4A).

Based on these observations, after the treatment five women who belonged to CST IV changed their CST: one obtained CST I, three were reassigned to CST III, and one was assigned to CST IV-C3. One individual who belonged to CST IV-C3 changed to CST IV and one subject who had CST III was reassigned to CST V. Data were summarised using these dynamics in the alluvial plot in Figure 4B.

**Figure 5:**
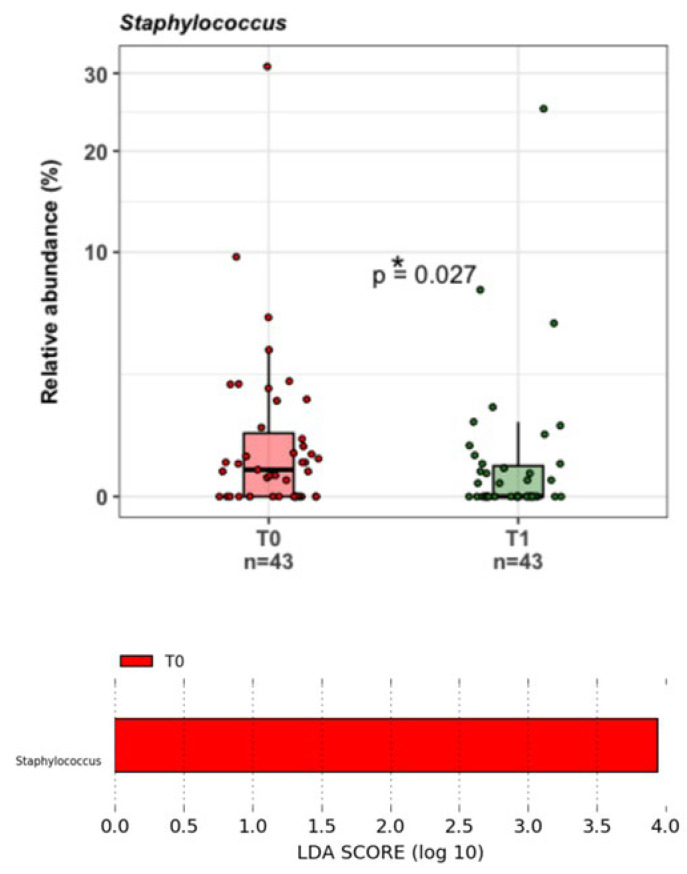
LDA LEfSe barplot displaying the different associations of bacterial genera between untreated (T0, in red) and treated menopausal women (T1, in green); the latter is not visible in the panel since the comparison demonstrated a significant association only at T0 (LDA score > 2.0). Box-and-whisker plots with data points show the relative abundances of the *Staphylococcus* genus in the two groups. Mann–Whitney U Test result of the group comparison is shown in the figure. * *p* < 0.05.

Significant microbial taxa signatures pre- and post-treatment were evaluated using the LDA LEfSe algorithm (https://huttenhower.sph.harvard.edu/galaxy/; accessed on 22 December 2023), and the genus *Staphylococcus* was found to be negatively associated with T1 (Figure 5).

### 3.2. VHI

**Figure 6:**
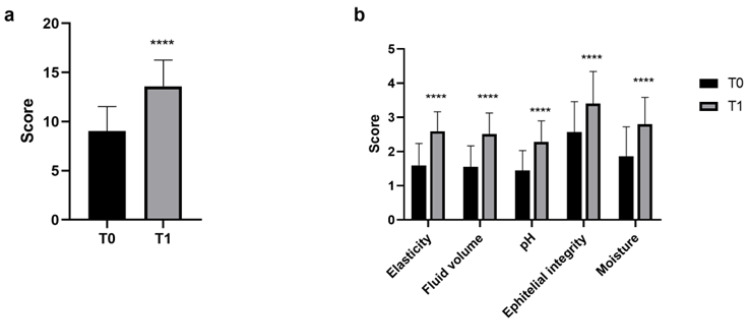
(**a**) Total VHI score comparison between T0 and T1. (**b**) A single score of the parameters is included in the VHI evaluation. Results are expressed as mean ± SD. **** *p* < 0.0001.

The mean initial VHI score of the enrolled women was <10, indicating the presence of vaginal discomfort. Although a score > 15 was not achieved, a 50% improvement in the VHI after only 28 days of probiotic treatment was observed (T1). The difference between T0 and T1 was statistically significant with a score of 9.02 ± 2.50 versus 13.59 ± 2.66, respectively (*p* < 0.0001; Figure 6a).

A significant improvement in all parameters analysed was observed, mainly in vaginal elasticity and secretion with a 60% score increase (*p* < 0.0001; Figure 6b).

**Figure 7:**
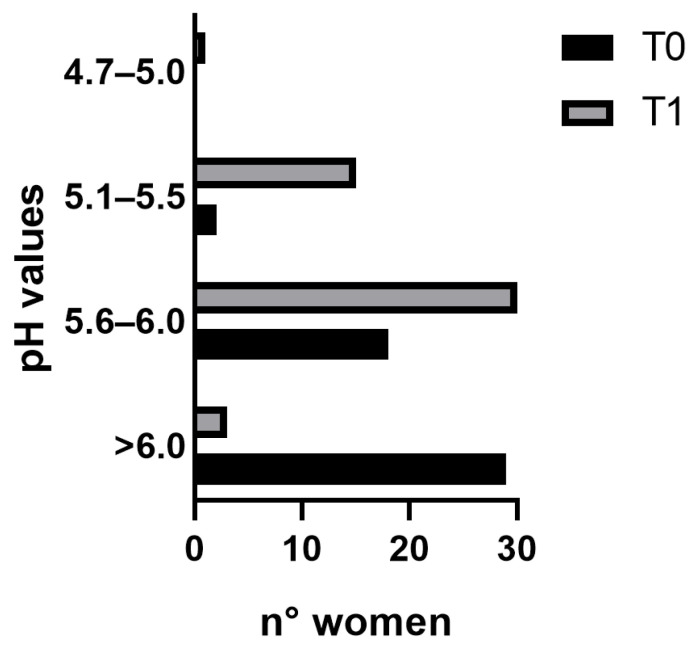
pH ranges of the vaginal environment of women enrolled at the beginning of the study (T0) and after 4 weeks of probiotic supplementation (T1).

In particular, pH values of the vaginal environment at T0 were mostly > 6.0 (29 out of 49 subjects), while at T1 a reduction was observed. Indeed, the majority of the women after the probiotic treatment had acidic pH values with respect to T0, ranging from 5.1 to 6.0 (Figure 7).

### 3.3. Cytokine Concentrations

The concentration of vaginal cytokines was measured in all 98 vaginal swabs, collected at enrollment (T0) and after 28 days of probiotic supplementation (T1), to explore the changes in local immune response after probiotic treatment. The inflammatory profile was assessed by the detection of the pro-inflammatory cytokines IL-6, IL-1β, TNF-α, and IL-8.

**Figure 8:**
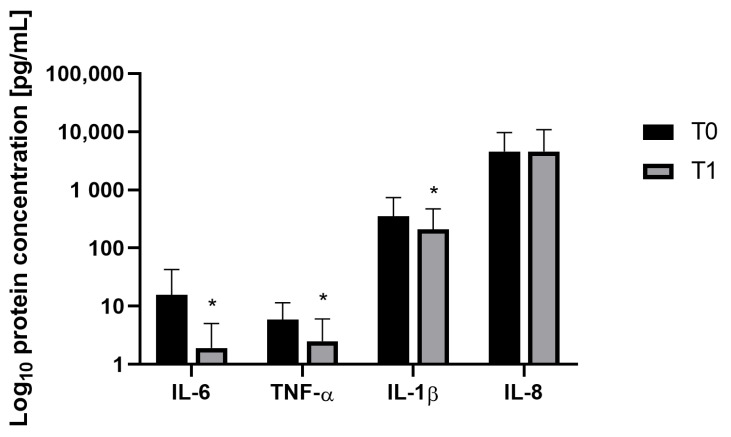
Vaginal concentrations of the interleukins were investigated in this study, comparing the conditions at the beginning of the study (T0) and after the probiotic treatment (T1). Results are expressed as mean ± SD. * *p* < 0.05.

The mean concentration of IL-6 decreased at T1 from 15.65 to 1.91 pg/mL, indicating an 87.80% improvement in the inflammation pattern. The mean concentration of TNF-α changed from 5.87 to 2.49 pg/mL with an amelioration of 57.6%; the mean value of IL-1β reduced from 351.1 to 208.02 pg/mL, resulting in a 40.8% improvement. The improvement of IL-6, TNF-α, and IL-1β were statistically significant (*p* < 0.05; Figure 8).

No significant difference was observed for IL-8, which remains almost unchanged from T0 to T1 (4.59 and 4.56 ng/mL, respectively; Figure 8).

## 4. Discussion

The transition into menopause is a natural biological process followed by hormonal, physical, and psychological changes that significantly affect a woman’s health and well-being [38,39]. The intricate interplay of hormonal fluctuations, primarily the decline in oestrogen levels, underlies the multitude of symptoms and health-related consequences experienced during the menopausal transition, such as hot flashes, night sweats, vaginal dryness, and alterations in mood [40]. Beyond the realms of reproductive health, menopause has been linked to alterations in bone density, cardiovascular health, cognitive function, and metabolic processes, raising critical concerns regarding the long-term health implications and disease susceptibilities among menopausal women [41]. Currently, the management of menopausal symptoms predominantly revolves around HRT, lifestyle modifications, and non-hormonal interventions [42].

Emerging research has shed light on the intricate relationship between menopause and alterations in the vaginal microbiota (VMB). The microbial composition in the vagina stands apart from other human surfaces and mucosal areas due to its lower microbial variety, mainly governed by *Lactobacillus* species [43]. These bacteria, which acidify the vaginal environment, have a crucial role in local defence [44,45]. The lowering of oestrogen and glycogen levels induces the thinning of the vaginal epithelium and the shifting of the VMB from a Lactobacilli-enriched ecosystem to an opportunistic pathogen infection-prone environment (CST-IV). Therefore, it coincides with a rise in pH, reduced vaginal secretion, dryness, and dyspareunia together with a dysbiotic vaginal milieu mainly composed of a higher proportion of anaerobic bacteria (e.g., *Bacteroides mimicus*, and *Mobiluncus motile curvilinearis*) and vaginosis-related bacteria, such as *G. vaginalis* [15].

The oral intake of probiotics allows the restoration of the functional activities of the gut microbiota. Moreover, they may reach the vagina, colonise it, and have long-lasting positive effects [46,47]. In light of common procedures to mitigate menopausal symptoms, the potential role of probiotics offers an alternative through their ability to restore and maintain healthy vaginal microbiota.

The aim of this study was to assess whether a supplement containing *L. plantarum* PBS067, *L. rhamnosus* LRH020, and *B. animalis* subsp. *lactis* BL050 could provide safe support to postmenopausal women, especially in rebalancing their vaginal microbiota. These specific strains were previously tested in two different 3D tissue models to investigate their capability to adhere, colonise, and inhibit urogenital pathogen growth. In the reconstructed human vaginal and bladder epithelia, they reduced the growth of *Candida glabrata*, *Neisseria gonorrhoeae*, *Trichomonas vaginalis*, and *E. coli*. Their mechanism of action not only includes the formation of a probiotic monolayer and an acidic microenvironment which prevents pathogen adhesion, but also the co-aggregation with *G. vaginalis*, *E. coli*, and *C. albicans*, causing the inhibition of pathogen cell growth. This evidence was demonstrated for each strain and its mixture [11].

The results collected, thanks to NGS analysis, highlight the effect of this probiotic composition in reducing the abundance of pathogenic bacterial genera like *Staphylococcus*. This bacterial genus harbours species involved in UTIs like *Staphylococcus epidermidis*, *S. saprophyticus*, and *S. aureus*, or in AV which are predominately associated with *S. aureus*, *Escherichia coli*, and *Streptococcus agalactiae* [48,49,50,51].

In this clinical trial, after probiotic treatment, five vaginal ecosystems transitioned from CST IV to CST III or CST I or CST IV-C3; only one vaginal microbiota passed from CST IV-C3 to CST IV, showing an inverse behaviour. Taken together, these data highlight that the probiotic treatment did not significantly change the vaginal environment. However, when it did occur, this was by a gain of a Lactobacilli or Bifidobacteria taxa, with the exception of only one subject. Based on these results, the treatment did not show any adverse effects on the vaginal microbiome composition or structure. Moreover, it seemed to be exerting a protective action against the presence of the *Staphylococcus* genus. By looking at the mean RA% of *Lactobacillus* and *Bifidobacterium* genera, *Lactobacillus crispatus*, *L. iners*, and *L. jensenii*, we noticed an increase in the mean values between T0 and T1. Although these increases were not significant, they described a trend that should be further investigated, for example, with a longer probiotic treatment and/or in a bigger cohort of women.

Moreover, an increase in the VHI score, as well as each single parameter, was observed. In particular, an improvement of 51% in the VHI after just 28 days of treatment occurred with respect to the beginning of the study. Several articles report a vaginal well-being VHI cut-off value set at 15; however, this value is linked to women of childbearing age [35,52]. It is known that during menopause, physiological changes lead to increased vaginal issues affecting vaginal mucosa well-being [35]. To the best of our knowledge, we have not found a defined and validated cut-off for menopausal women. Nevertheless, the results obtained within just 28 days of product intake demonstrated a significant improvement, not only in overall vaginal health but also in various subcategories, displaying a considerable increase compared to the baseline condition. Specifically, a greater increase of 60% was observed concerning elasticity and fluid volume, indicating improved vaginal lubrication, and consequently a reduced atrophy and inflammatory status [53,54]. Similarly, mucosal hydration and vaginal epithelium integrity showed a respective score increase of 50% and 33%. It is important to note that the women enrolled did not exhibit any critical conditions at the time of recruitment or throughout the study’s duration. Particular attention should be given to the pH modification closely linked to the presence of Lactobacilli. Sarmento and colleagues observed that values below 6 are uncommon in menopausal women [34]. This condition negatively affects the vaginal environment, heightening the risk of bacterial infections among women. The results of this study highlighted that a 58% reduction in the pH value correlated with an increase in lactic acid production. Specifically, it can be noted that at T0 nearly 60% of the women had a pH > 6.0, a condition that decreased to 6% after 28 days of treatment. It is also noteworthy to observe changes in other pH ranges after probiotic intake. Notably, there was a shift from 61% to 36% in the range between 5.6 and 6 and from 31% to 4% in the range between 5.1 and 5.5, between T1 and T0, respectively.

Additionally, vaginal swabs were collected to determine vaginal inflammatory status. A significant amelioration of the inflammatory pattern was observed. In this study, the inflammatory profile was assessed by measuring levels of four pro-inflammatory cytokines, IL-6, IL-1β, TNF-α, and IL-8, known to be associated with inflammatory processes linked to vaginal infections. Increased concentrations of pro-inflammatory cytokines in post-menopausal women contribute to the susceptibility to chronic inflammatory diseases [55]. Recent evidence has demonstrated that Lactobacilli possess immunostimulatory properties that aid in preventing and treating inflammatory bowel disease, mucositis, and even colon cancer effectively [56]. Recent scientific findings suggest that utilising LAB directly in the vagina and orally as adjunct therapy could serve as a promising approach to rejuvenating the vaginal microbiota in menopausal women through distinct modes and mechanisms [57].

Post-treatment, the average concentration of IL-6 decreased significantly, indicating a remarkable 87.80% reduction in inflammation. Similarly, the average TNF-α level dropped, denoting a 57.60% decrease, while IL-1β showed a statistically significant 40.80% reduction (*p* < 0.05). No significant variation was observed for IL-8, maybe due to the absence of ongoing infections [58].

Given the importance of the vaginal microbiota in women’s health and the limited understanding of AV, exploring the vaginal microbiome and its correlation with local immune factors offers a chance to establish the connection between vaginal beneficial microorganisms and potential pathogens concerning specific vaginal CST [59]. Moreover, this study explores the possibility of using this inexpensive and well-tolerated probiotic treatment as a preventive oral intervention in a peculiar moment of a woman’s life not deeply investigated, like post-menopause, giving beneficial effects for their overall well-being.

## 5. Conclusions

Studies investigating the direct effects of probiotic interventions on the vaginal microbiota composition and associated genitourinary symptoms in menopausal women are notably limited. Strategies aimed at restoring and maintaining a healthy vaginal microbiota could potentially mitigate genitourinary symptoms and reduce the risk of associated infections. Further exploration of probiotic interventions may offer promising avenues for managing vaginal dysbiosis during menopause, thereby enhancing the overall quality of life for post-menopausal women. In this context, the presented data aim to delve into the multifaceted aspects of menopause, exploring its physiological underpinnings, associated symptoms, and broader implications for women’s health. Our results showed that a combination of *L. plantarum* PBS067, *L. rhamnosus* LRH020, and *B. animalis* subsp. *lactis* BL050 can positively modulate the inflammatory profile typical of post-menopause, restore vaginal microbiota, and relieve vaginal distresses.

The study limitations comprise the absence of a placebo group. This omission was deliberate to focus solely on evaluating the probiotic composition effectiveness, comparing every subject pre-and post-treatment. However, future research including a placebo arm could provide a more comprehensive understanding of its comparative efficacy. Additionally, the duration of our clinical trial was relatively short. While it provided valuable insights into short-term effects, a longer trial duration would offer a more comprehensive understanding of the treatment’s efficacy.

## Figures and Tables

**Table 1:** Inclusion and exclusion criteria of the study.

Inclusion Criteria	Exclusion Criteria
Post-menopausal women for 18 months (last menstruation observed)	Women undergoing HRT
Women with BMI ≤ 27	Women with BMI > 27
Women with vaginal pH ≥ 5	Women with a demonstrated allergy to one or more ingredients contained in the product
Menopausal women with typical menopausal complaints such as burning, redness, stinging sensation, oedema, dyspareunia, and discharge	Women who intend to use probiotic products (e.g., yoghurt fortified with probiotics or probiotic-based dietary supplements) and have used them in the past 2 weeks
Women who intend to use the probiotic product and undergo follow-up visits	Women who have undergone antimicrobial treatment within the past 4 weeks
Post-menopausal women for 18 months (last menstruation observed)	Women undergoing HRT
Women with BMI ≤ 27	Women with BMI > 27
Women with vaginal pH ≥ 5	Women with a demonstrated allergy to one or more ingredients contained in the product

**Table 2:** VHI parameters and scores to assess vaginal hydration and secretions, elasticity and appearance of the vaginal mucosa, and detection of vaginal pH.

Score	Overall Elasticity	Fluid Secretion Type and Consistency	pH	Epithelial Mucous	Moisture
1	None	None	>6.0	Petechiae noted before contact	None, mucosa inflamed
2	Poor	Scant, thin yellow	5.6–6.0	Bleeds with light contact	None, mucosa not inflamed
3	Fair	Superficial, thin white	5.1–5.5	Bleeds with scraping	Minimal
4	Good	Moderate, thin white	4.7–5.0	Not friable, thin mucous	Moderate
5	Excellent	Normal (white flocculent)	<4.6	Not friable, normal mucosa	Normal

## Data Availability

The NGS data presented in this study are openly available on the Sequence Read Archive (SRA) with project reference number PRJNA1054360.
